# Selective Laser Sintering of Solid Oral Dosage Forms with Copovidone and Paracetamol Using a CO_2_ Laser

**DOI:** 10.3390/pharmaceutics13020160

**Published:** 2021-01-26

**Authors:** Yanis A. Gueche, Noelia M. Sanchez-Ballester, Bernard Bataille, Adrien Aubert, Laurent Leclercq, Jean-Christophe Rossi, Ian Soulairol

**Affiliations:** 1 ICGM, University Montpellier, CNRS, ENSCM, 34000 Montpellier, France; yanis-abdelhamid.gueche@etu.umontpellier.fr (Y.A.G.); noelia.sanchez-ballester@umontpellier.fr (N.M.S.-B.); bernard.bataille@umontpellier.fr (B.B.); adrien.aubert@umontpellier.fr (A.A.); 2 IBMM, University Montpellier, CNRS, ENSCM, 34000 Montpellier, France; laurent.leclercq@umontpellier.fr (L.L.); jean-christophe.rossi@umontpellier.fr (J.-C.R.); 3 Department of Pharmacy, Nîmes University Hospital, 30900 Nimes, France

**Keywords:** 3D printing, selective laser sintering, copovidone, oral dosage forms, material suitability, printability

## Abstract

Material suitability needs to be considered for the 3D printing of solid oral dosage forms (SODFs). This work aims to assess the suitability of a CO_2_ laser (λ = 10.6 μm) for selective laser sintering of SODFs containing copovidone and paracetamol. First, physicochemical characterization of powders (two grades of copovidone, two grades of paracetamol and their mixtures at various proportions) was conducted: particle size distribution, morphology, infrared absorbance, flowability, and compactness. Then, printing was launched, and printability of the powders was linked to their physicochemical characteristics. The properties of the sintered SODFs were evaluated (solid state, general aspect, porosity, hardness, drug content and release). Hence, it was found that as copovidone absorbs at the laser’s wavelength, sintering was feasible without using an absorbance enhancer. Also, flowability, which mainly depends on the particle size, represents the first control line for “sinterability” as a fair flow is at least required. Low compactness of copovidone and mixtures reduces the mechanical properties of the SODFs but also increases porosity, which can modulate drug release. Moreover, the drug did not undergo degradation and demonstrated a plasticizer effect by lowering the heating temperature. In conclusion, this work proves the applicability of CO_2_ laser SLS printer to produce SODFs.

## 1. Introduction

3-Dimensional printing is set to be the next technology that will revolutionize the pharmaceutical industry in the coming years. In a time where personalized medicine is gaining more and more ground, additive manufacturing (AM) could be an interesting solution to tailor drugs to meet the personal needs of each patient. This state of the art technology has already gone beyond the stage of simple experimentation as the commercialization of Spritam^®^, the first FDA-approved 3D printed pill, proves it [1]. Among the diverse techniques of AM, fused deposition modeling (FDM) seems the most promising one, within the scientific community. Indeed, the production of solid oral dosage forms (SODFs) by FDM has been the subject of 72 papers from 2014 to 2019 [2].

**Figure 1:**
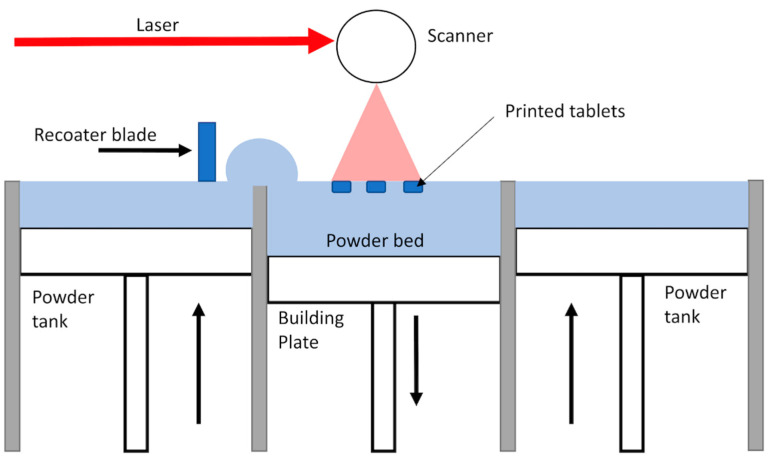
Schema of the Selective Laser Sintering process.

On the other hand, other 3D printing techniques remain not profoundly explored for pharmaceutical applications, for example, Selective Laser Sintering (SLS), which also proved to be very attracting [3,4] and might outweigh FDM in terms of precision and applied temperatures. This technique consists of the consolidation of powder particles with the energy provided by a laser. The process of SLS starts by the spreading of a thin layer of powder over the building area. Then, the laser scans the powder bed according to a specific pattern dictated by the pre-established design of the object, to fuse partially or completely the particles depending on the amount of transmitted energy. Next, the build platform lowered, and another layer of powder is spread over the previously sintered layer. This process repeats itself until complete achievement of the object (Figure 1). The powder that has not been consolidated remains in place and serves as a support to the object during its building. Powder can also be recycled after sieving, making the SLS a very economical technique [5]. One of the main advantages of the SLS for drug manufacturing along its high resolution is that the feedstock is powder, which is common to other pharmaceutical manufacturing processes. Hence, there is no need to pretreat the material like in FDM in which filaments need to be produced by Hot Melt Extrusion (HME) [6].

To date, although only a few papers related to the production of SODFs by SLS have been issued [7,8,9,10,11,12,13,14,15,16,17,18,19,20], they demonstrated clearly that it is possible to manufacture oral medicines with a sintering machine using different pharmaceutical thermoplastic polymers (copovidone, cellulose derivatives and Eudragit^®^). These contributions distinctly highlight the most important benefit that SLS could have in pharmaceutical manufacturing: its ability to create more or less porous forms by modulating the printing parameters and hence control the drug release from the printed SODFs.

It is important to note that although all the aforementioned work was conducted with SLS machines that use a blue diode laser, none of the evaluated polymers absorbed at the wavelength of the laser. Therefore, a colorant e.g., “Candurin^®^” was added to enhance the absorbance and allow the sintering process [7,8,9,10,11,12,13,14,15,16,17,18,19,20]. Nonetheless, the majority of commercially available SLS printers use a different laser beam, which is the carbon dioxide (CO_2_) laser beam (λ = 10.6 μm). This laser is relatively powerful and could be detrimental to active ingredients. However, incorporation of an absorbance enhancer could not be required since many biocompatible and biodegradable polymers absorb at the wavelength region of the CO_2_ laser, as demonstrated by Salmoria et al. [21]. This research team has printed drug delivery devices (DDDs) with a CO_2_ laser using different polymers such as polycaprolactone [22] and polyethylene [23]. However, none of the studies conducted with the CO_2_ laser on DDDs, evaluated drug stability [4]. Hence, the implementation of CO_2_ laser SLS for the production of oral dosage forms, would need to overcome the barrier of drug degradation. A way to achieve this is to use SLS printers that can modulate the laser power, a non-modifiable parameter in the commonly used Sintratec^®^ Kit printer.

Furthermore, the design and development of oral medicines by 3D printing techniques requires suitable material for this purpose. Printability of pharmaceutical polymers for FDM has been the subject of many articles in the last years. For example, it has been demonstrated that drug loading can affect the brittleness of the extruded filament and therefore may induce clogging during FDM printing [24,25]. As for SLS, except for a verification of the polymer’s absorbance at the laser’s wavelength [7], the relationship between the properties of pharmaceutical polymers and sintering process remains not profoundly investigated. In order to be “sinterable”, the polymeric material should present suitable physicochemical properties. The adequate physical properties include good flowability and high packing density (compactness), which are mostly influenced by the granulometric and morphologic characteristics of the powder particles. In addition, the polymer should absorb at the laser’s wavelength which is mainly dependent on its chemical structure. Good flowability is desirable to achieve an effective powder deposition whereas compactness and absorbance are known to control the subsequent laser consolidation [5,26]. Although critical attributes for sintering are relatively well understood [27], to our knowledge, there is no report of using pre-sintering methods to allow a fast screening of printable pharmaceutical polymers and formulations. Such tools could be beneficial for future pharmaceutical research to choose suitable powders for SLS and avoid trial-and-error methods. Far more importantly, study of printability could give more insight into the pharmaceutical materials’ sensitivity towards sintering and help manufacturers to improve the properties of already available thermoplastic pharmaceutical polymers that may be suitable for HME processes but not for SLS. Also, important to note that SLS, as well as other 3D printing techniques, show an important advantage in terms of personalizing oral dry forms and are therefore more intended for precision medicine than mass manufacturing. SLS is only at its beginning in the pharmaceutical landscape and more printability studies to implement the technology at a clinical scale and industrial scale will be required.

Kollidon^®^ VA64 (copovidone) is a water-soluble and thermoplastic copolymer composed of hydrophilic vinylpyrrolidone and lipophilic vinyl acetate. It is broadly used in pharmaceutical applications such as binder in the production of granules by wet granulation, dry binder in direct compression, film former in tablet coating, and polymeric matrix in HME [28]. Kollidon^®^ VA64 was previously investigated as a polymer backbone in FDM and proved to be beneficial in terms of lowering the process temperatures and accelerating the drug release [29]. It has also been tested on SLS and particularly for the production of orally disintegrating tablets (ODTs) [8,14,15]. Moreover, Kollidon^®^ VA64 is available in two different grades depending on the particle size. This could be interesting for SLS since the particle size distribution is the main aspect to take into consideration, as mentioned above.

The aim of this work is to assess the suitability of a CO_2_ laser (λ = 10.6 μm) for selective laser sintering of oral dosage forms using Kollidon^®^ VA64 as a polymeric carrier and Paracetamol as a model drug. Prior to sintering, native powders, as well as mixtures, were characterized in order to understand the thorough relationship between their physicochemical properties and printability. An SLS commercial powder “polyamide 12” was chosen as a reference material in order to interpret the results for Kollidon^®^ VA64 and the powder mixtures. Then, printed solid oral dosage forms were characterized, and drug stability was studied by ultra-high performance liquid chromatography (UHPLC). Finally, the influence of drug loading on both the sintering process and the properties of the printed SODFs was assessed.

## 2. Materials and Methods

Kollidon^®^ VA64 (KVA64) and Kollidon^®^ VA64 Fine (KVA64F) were generously donated by BASF (Ludwigshafen, Germany). Duraform^®^ polyamide 12 (PA12) was provided by 3D Systems (Santa Clarita, CA, USA) and used as a reference powder. Paracetamol crystal (PAR) and paracetamol crystal fine (PAR F) were purchased from Sequens (Porcheville, France).

### 2.1. Physicochemical Characterization of Powders

#### 2.1.1. Scanning Electron Microscopy (SEM)

In order to study the particle morphology of powders, images of each of KVA64, KVA64F, PA12, PAR and PAR F were taken with a scanning electron microscope (4800 S, Hitachi, Tokyo, Japan) after platinum sputtering under vacuum before observation. The microscope was also used to take images of the printed SODFs (surface and vertical sections).

#### 2.1.2. Preparation of Mixtures

Mixtures of the two grades of copovidone were prepared at three different proportions (Table 1). Formulations based on KVA64 and paracetamol (PAR and PAR F) were also prepared at three different drug loadings (Table 1). Mixing was conducted on a 3D shaker mixer Turbula^®^ T2F (WAB, Muttenz, Swizterland) at a speed of 49 rpm for 10 min.

#### 2.1.3. Laser Granulometry

Dry laser diffraction (Mastersizer 2.18, Malvern Instruments Ltd., Malvern, UK) was used to determine the mean particle size (D (4,3)) and the size distribution (span = ((D90 − D10))/D50) of the native powders as well as the mixtures. A jet pressure of 1.2 bar was used to deagglomerate the particles during laser measurement. Data treatment was realized using the software Mastersizer S 2.18 and choosing the analysis mode as polydisperse. For each powder, a sample of approximately 1 g was analyzed, and each measure was performed at least in triplicate.

#### 2.1.4. Study of Flowability and Compactness

Compactness of the native powders and the mixtures was assessed by the measurement of bulk (*BD*) and tapped density (*TD*). The test was conducted following the method described in the European Pharmacopeia [30] with a 250 mL graduated cylinder and a sample mass of 30 g. Hausner ratio (*HR*) was then calculated to express the powder flowability, according to the following formula:

(1) $$HR=\frac{TD}{BD}\;$$

Powder flowability was also evaluated by the measurement of the angle of repose (AOR) using a granulate flow tester GTB (Erweka, Langen, Germany) according to the European Pharmacopeia guidelines [31]. It was conducted by allowing a mass of 30 g of each powder positioned above a fixed diameter base to drain from a 200 mL funnel through a 15 mm nozzle. Stirring was fixed at the speed 4. The drained angle of repose was determined from the cone of powder formed on the base. Each measure was done in triplicate.

#### 2.1.5. Fourier-Transform Infrared Spectroscopy (FTIR)

Infrared spectrophotometer Vector 22 FTIR (Bruker, Billerica, MA, USA) was employed to evaluate the absorbance of the polymers (KVA64 and PA12) as well as paracetamol at the wavelength of the printer’s laser. Absorbance was recorded from 4000 to 400 cm^−1^ at room temperature (approximately 25° C) and 64 scans were averaged at a resolution of 4 cm^−1^. Samples of 100 mg were prepared by blending 10 mg of the polymer or 1 mg of the drug with Q.S. (*Quantum satis*) of anhydrous potassium bromide (previously dried in the oven at 100 °C for 30 min) and compressing the mixture to form a disk. The FTIR spectrums were treated using the infrared software OPUS 6.5 (Bruker, Billerica, MA, USA).

### 2.2. Printing of SODFs:

3D model of a cylindrical dosage form (10 mm diameter and 3 mm height) was designed using an online CAD software OnShape^®^ (Onshape, Boston, MA, USA) and exported as a STL file. Then, it was converted to a G-code with an open source software Slic3r^®^ 1.2.9 before transferring it to the 3D SLS printer Sharebot^®^ SnowWhite (Sharebot, Nibionno, Italy).

A mass of 300 g from each powder was loaded in the reservoir tanks and the building platform (100 × 100 × 100 mm^3^) to the brim. The air gaps formed in the deposited powder were eliminated by recalibrating the level of the tanks. An automatic recoater blade removed the surplus of powder on top of the building platform to create a flat surface. For the case of powders with poor flowability, powder filling, recalibrating, and recoating were repeated until the formation of a flat surface. For all the printings, the temperature mode was set at “powder temperature” which meant that the heaters were controlled by the temperature of the powder bed.

The optimized printing parameters for polyamide 12 were previously developed in our department. For the other printable powders, an optimized setting (Table 2) for which all the SODFs were completely printed (with no missing layer), was achieved after preliminary tests. Heating temperature (°C), laser power (% of the maximum laser power) and scan speed (pps or points per second ≈ 0.05 mm/s) were machine parameters, whereas the layer thickness (mm) was entered in Slic3r^®^.

Thirty-six SODFs were launched for printing per batch. The process started with the heating of the powder by infrared lamps (230 W) for thirty minutes. Afterward, a CO_2_ laser (14 W) sintered the successive powder layers according the 3D model of SODFs. The overall printing time depended mainly on the chosen scan speed and layer thickness. Finally, when printing was completed, the powder bed containing the printed SODFs was removed and sieved using a 250 μm sieve to eliminate the excess powder around the SODFs.

### 2.3. Characterization of the Printed SODFs

#### 2.3.1. Differential Scanning Calorimetry (DSC)

DSC was used to determine the melting point (or the glass transition temperature) and the solid state of polymers, drug, physical mixtures and printed SODFs. Accurately weighed samples (5–10 mg) were placed in sealed aluminum pans and heated from 25 °C to 200 °C at 10 °C/min with a DSC 4000 (Perkin Elmer, Waltham, MA, USA). A heat-cool-heat cycle method was conducted to remove the thermal history of copovidone. Nitrogen was used as a purge gas with a flow rate of 20 mL/min. Data collection and analysis were conducted using Pyris Manager software (Perkin Elmer, Waltham, MA, USA).

#### 2.3.2. X-ray Powder Diffraction (XRPD)

The solid state of the polymers, drug, physical mixtures and printed SODFs was characterized using a Bruker D8 Advance diffractometer (Bruker, Billerica, MA, USA) and the monochromatic Cu Kα1 radiation (λα = 1.5406 Å, 40 kV and 40 mA). The angular range of data recording was 2–80° 2θ, with a stepwise size of 0.02° and a speed of 0.1 s counting time per step, using LYNXEYE detector 1D (Bruker, Billerica, MA, USA).

#### 2.3.3. Weight, Dimensions and Mechanical Strength of the Printed SODFs

Weight of the SODFs was determined using a precision electronic balance Adventurer^®^ (OHAUS, Parsippany, NJ, USA). Physical dimensions (height and diameter) and hardness were measured using a Sotax Multitest 50FT (Sotax AG, Basel, Switzerland). Measurements were carried out on 10 SODFs per printing batch and results were expressed as the mean value ± standard deviation.

#### 2.3.4. Disintegration Time of the Printed SODFs

Disintegration tests were performed on a disintegration apparatus (Sotax DT50, Sotax AG, Basel, Switzerland) with distilled water at 37 °C according to the European Pharmacopeia guidelines [32]. For each printing batch, six SODFs were tested simultaneously. The disintegration time was reached when no residues were present on the bottom of the test basket. Results were reported as the mean value ± standard deviation.

#### 2.3.5. Drug Content of the Printed SODFs

For each formulation, three individual SODFs were dissolved in 100 mL of distilled water. Samples of the solutions were then diluted and the drug concentration was determined by ultra-high performance liquid chromatography (UHPLC, Thermofisher Scientific, Waltham, MA, USA) using a UHPLC-DAD system. It consisted of a Thermo Scientific™ Dionex™ UltiMate™ 3000 BioRS equipped with a WPS-3000TBRS autosampler and a TCC-3000RS column compartment set at 35 °C. The system was operated using Chromeleon 7 software (Thermofisher Scientific, Waltham, MA, USA). An Accucore C18 column (2.6 µm, 100 × 2.1 mm^2^) combined with a security guard ultra-cartridge (Phenomenex Inc., Torrance, CA, USA) was used. An isocratic binary solvent system was utilized, consisting of water/formic acid (1%, *v*/*v*) as solvent A and acetonitrile/formic acid (1%, *v*/*v*) as solvent B (90%A, 10%B). The flow rate of the mobile phase was 1.5 mL/minute, and the injection volume was 50 μL. Quantitative analysis of paracetamol in the SODFs was carried out using an external standard method. The calibration curve was constructed using 5 different standard levels in the concentration range 1–20 mg/L. The peak of paracetamol was monitored at 244 nm.

#### 2.3.6. Size Exclusion Chromatography—Multi Angle Light Scattering (SEC-MALS)

The degradation of copovidone during the SLS process was assessed by analyzing both the raw polymer and the sintered KVA64 placebo SODF on SEC-MALS (Thermofisher Scientific, Waltham, MA, USA). The experiments were performed at 35 °C on a Thermo Scientific Ultimate 3000 module equipped with a OHpak SBG Shodex column guard (50 × 6 mm^2^) and a SB-805-HQ Shodex column (300 × 8 mm^2^) connected in series in association with a miniDawn Treos laser light scattering detector having a 658-nm laser (Wyatt Technology Corp., Santa Barbara, CA, USA) and with a RID-6A refractive index monitor (Shimadzu Corp., Kyoto, Japan). The eluent used was composed of a mixture of 0.15 M phosphate buffer and 1 M NaCl at pH = 7.4. The eluent was filtered using Durapore membrane filters of 0.1 μm cut-off. Incremental refractive index (dn/dc) value of 0.15 was used, as found in the literature [33]. The polymer samples (100 μL injection volume at a concentration of 1 g.L^−1^) were eluted at a 1 mL.min^−1^ flow rate. The data were analyzed using the Astra software (Wyatt Technology Corp., Santa Barbara, CA, USA, v6.1.1.17).

#### 2.3.7. Drug Release of the Printed SODFs

A dissolution test was carried out for SODFs containing paracetamol with a Pharma Test DT70 dissolution tester (Pharma Test Apparatebau AG, Hainburg, Germany) using a paddle apparatus (European Pharmacopeia) [34]. For each formulation, three SODFs were randomly selected and individually placed in the dissolution vessels, each containing 900 mL of 0.1 M HCl (sink condition) and stirred at 100 rpm and 37 ± 0.5 °C. Samples were withdrawn automatically each 2 min and analyzed using a continuous flow through system attached to an 8 cell UV/Vis spectrophotometer Specord 250 (Analytik Jena, Jena, Germany) at a wavelength of 268 nm. Results were expressed as mean values with standard deviation.

## 3. Results & Discussion

### 3.1. Physicochemical Characterization of Powders

To achieve successful powder deposition and effective object densification in SLS, feedstock material should exhibit smooth flowability and good compactness. These properties are mainly governed by the shape and the particle size distribution of the particles [35]. It also seems necessary for the material to absorb at the wavelength of the laser beam so that it can acquire thermal energy for sintering [36].

Hence, prior to sintering, the physicochemical properties of the pharmaceutical polymers (particle shape, size distribution, flowability, and compactness) were studied to assess their suitability for sintering and choose the appropriate material (KVA64, KVA64F or mixture of both grades) for the printing process. Formulations prepared with two grades of paracetamol were also evaluated to understand the impact of the drug’s physicochemical characteristics on printability. Polyamide 12 was taken as a reference material. This type of nylon is by far the most widely used SLS polymer due to its historically recognized processability and relatively low cost compared to other materials [37]. This approach was already used [38] when filaments made with pharmaceutical polymers were compared to commercial filaments in terms of mechanical properties to predict their feedability on FDM.

#### 3.1.1. Particle Morphology and Size Distribution

**Figure 2:**
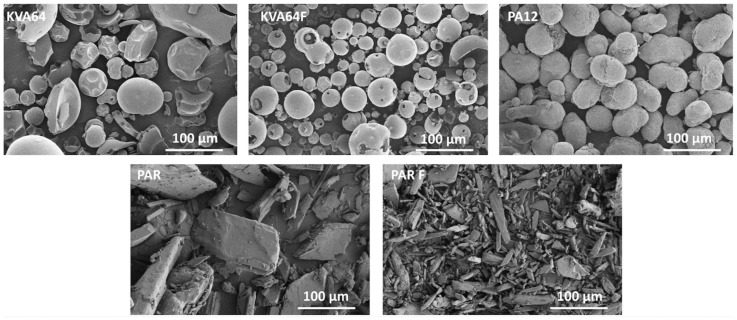
SEM images for KVA64, KVA64F, PA12, PAR, and PAR F powders.

The observation of particle morphology by SEM (Figure 2) showed that copovidone is in the form of hollow smooth spheres, more or less fragmented for both grades. On the other hand, polyamide 12 is composed of filled oval-shaped particles. Both grades of paracetamol present particles with irregular morphologies. PAR particles are large and plate-like whereas PAR F particles are thin and needle-like. In SLS, spherical particles are highly recommended to improve both rheological performance [39] and packing behavior [40], which is in favor of copovidone particles.

Laser granulometry conducted with no jet pressure (0 bar) revealed the presence of particles’ agglomerates, especially for KVA64F and PAR F and particle size was thus overestimated. At 2.4 bar, agglomerates were still present, and brittle particles such as PAR broke, which underestimated their particle size [41]. Therefore, an intermediate pressure of 1.2 bar, which simulates the normal conditions of powder handling and mixing, was chosen to disaggregate particles agglomerates with minimum breaking of the brittle particles. Laser granulometry (Table 3) revealed that KVA64 had a mean diameter of 71.5 μm compared to the fine grade, which presented a mean diameter of 26.0 μm. PA12 was found to have a mean diameter of 63.7 μm. Therefore, only PA12 and KVA64 were in the recommended particle size range (45–90 μm) for SLS [42]. Concerning the drug particles, PAR was over the recommended range (124.7 μm) and PAR F was below it (15.4 μm). It has been reported that coarse particles with high surface/volume ratio show poor compactness, but easy flow, whereas fine particles can expose better packing behavior but are difficult to handle as they have tendency to form very cohesive clusters due to the formation of high interparticular bonds [43]. Therefore, the optimal size should be intermediate, offering both good flowability and appropriate compactness. As for Particle Size Distribution (PSD); KVA64, KVA64F and PA12 had respectively a span value of 2.16, 2.16 and 0.93 (Table 3). Therefore, the PSD was narrower for the reference powder compared to copovidone. Moreover, the incorporation of increasing rates of KVA64F or PAR F powder in the KVA64 mixtures, tended to decrease the mean diameter and widened the distribution (Table 3). On the other hand, the introduction of PAR particles at rising percentages in the KVA64 mixture, increased the mean diameter and widened the PSD.

#### 3.1.2. Study of Flowability and Compactness

Bulk density was chosen as an indicator of compactness since no tapping nor compression was involved in the printing, but only a recalibration of the powder tanks was realized. Flowability was assessed by the determination of the Hausner ratio (HR) and confirmed by the measurement of the angle of repose (AOR). This latter technique was not applicable in all the cases due to the electrostatic character of copovidone, especially when fine particles were incorporated. Study of powder flowability and packing behavior (Table 4) evidenced the low bulk density (0.38 g/cm^3^) and the fair flow (HR = 1.25 and AOR = 37.7°) of KVA64. KVA64F is three times lighter (0.12 g/cm^3^) and has a very poor flow (HR = 1.57). On the other hand, PA12 is denser (0.48 g/cm^3^) and flows more easily (HR = 1.19 and AOR = 34.0). These results are not in agreement with previous studies stating that powders presenting high PSD are more compact and therefore exhibit higher values of bulk and tapped densities [44]. This indicates that PSD seems not enough to predict the packing behavior of powders.

Mixtures based on the two grades of Kollidon^®^ VA64 were prepared to obtain a more compact material by the effect of percolation of small particles between larger ones. This method derives from the Furnas Model [45] that aims to enhance powder packing density by adding fine particles of discrete diameters so they can pass through the voids of known sizes between the coarse particles. This approach generates a multimodal size distribution and effectively improves with a fine to coarse size ratio of 1/7 for spherical particles [46]. However, Kollidon^®^ VA64 blends exposed a Gaussian distribution (Supplementary data: Figure S1) and a size ratio of 1/3 between KVA64F and KVA64 (Table 3). Therefore, the improvement of compactness was not achieved, and bulk densities of mixtures were even lower.

Mixtures of KVA64 and PAR presented a similar bulk density (0.34 g/cm^3^) and were less dense than KVA64 alone. In general, mixtures of particles with different shape (spherical copovidone and irregular paracetamol) are known to generate more interparticular pores, which decreases compactness [47]. Interesting to note that although it was visible to the naked eye that those formulations presented acceptable flowability, the HR calculated indicated a poor or even very poor flow property. This can be explained by the fragmentation of the large brittle particles of PAR into smaller ones during tapping which overestimated the value of tapped density and Hausner ratio. The measured AOR increased with the percentage of PAR but the flow property remained fair for the three formulations which confirms the visual assessment. Moreover, incorporation of increasing amounts of PAR F to KVA64 reduced drastically the bulk density. The flow property was also highly affected reaching a HR value of 1.85 for the mixture 70% KVA64/30% PAR F due to the relatively low particle size of this grade of paracetamol.

Nevertheless, this conducted method to evaluate the rheological performance and the compactness of the feedstock lacks of accuracy because in a SLS printer the powder is rather spread over the printing bed layer by layer than deposited as bulk [26]. Consequently, this approach widely used for powders intended for tablet compression may only partially allow predicting the behavior of the powder in a sintering machine.

#### 3.1.3. Infrared Absorbance

**Figure 3:**
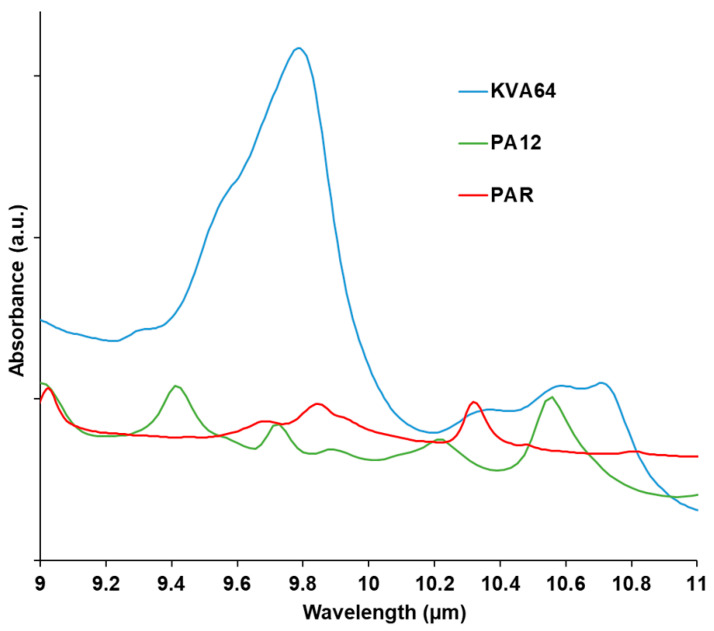
FTIR spectra of KVA64, PA12, and PAR from 9 to 11 µm.

Absorption of the polymer at the wavelength of the used laser has to be considered in SLS. The material should absorb enough so that it can acquire thermal energy. which allows the sintering process. On the other hand, absorption should not be excessive, otherwise, thermal degradation would occur [36]. Analysis of the FTIR spectra (Figure 3) at the region of the laser wavelength evidenced a broad halo of absorbance ranging from 10.2 to 11 µm for Kollidon^®^ VA64, and a more distinguishable peak at 10.6 µm for polyamide 12 as previously reported [48]. That region matches the fingerprint IR region (bending and stretching vibrations) in which the majority of the polymers absorb since they are constituted of aliphatic compounds (C-H).

Paracetamol does not absorb at the laser’s wavelength. Thus, no peak was observed at 10.6 µm (Figure 3). Therefore, the drug does not enhance the absorbance of KVA64 but rather decreases it when it is formulated with the polymer (Supplementary data: Figure S2).

### 3.2. Printing of SODFs

Although the physicochemical characterization demonstrated less favorable properties of copovidone for sintering than polyamide 12 (lower flowability, reduced compactness and less absorbance at the laser’s wavelength). The unique way to assess the “sinterability” of copovidone was to try the two different grades, mixtures, and formulations directly in the printer. Prior to sintering, the ability of the powders to form a flat layer on the first passage of the recoater blade was evaluated (Table 5). The powders that passed the test were: PA12, KVA64, mixtures of the two grades and the mixtures of KVA64 and PAR. As for KVA64F and the formulations of KVA64 and PAR F, they exhibited a non-flat layer of powder at the first try (Table 5). The formation of a flat layer was then achieved after filling the crevasses with powder and repeating the recoating. However, during the preheating phase and after multiple layer depositions, powders with passable flowability or less could not maintain the required flat layer. Because of the discontinuous layer of powder, the laser beam diffracted and did not sinter the particles according to the pre-established design. This demonstrates that poor flowability could hinder the sintering process at its first step by preventing the formation of a continuous layer of powder able to be sintered. From these results, it can be concluded that a Hausner ratio inferior to 1.25 and/or an angle of repose inferior to 40° seem to be necessary to achieve a proper powder deposition and ensure an effective printing on SLS. This recommended HR value has also also confirmed by an anterior study [49]. Thus, Hausner ratio and angle of repose seem to be very interesting tools for screening suitable pharmaceutical materials for SLS applications in the future. Important to highlight that these recommendations may be only applicable to this model of SLS machine and should be re-evaluated with other printers, especially equipment using a different spreading system such as a roller instead of a blade [50].

After several preliminary tests to set the optimal printing parameters (Table 2), each of KVA64, 90% KVA64/10% KVA64F, and the formulations prepared with PAR proved to be printable (Table 5).

Concerning the KVA64 absorbance, it was enough to ensure the sintering process. For the majority of polymers, absorbance enhances with increasing laser wavelength [36]. That explains the capacity of Kollidon^®^ VA64 to sinter with a CO_2_ laser (λ = 10.6 μm) and not with a blue diode laser (λ = 445 nm) [8]. In this study, the required energy density for sintering was higher for the pharmaceutical polymer (scan speed at 25,000 pps) than for the reference material (scan speed at 45,000 pps) (Table 2). The energy density (amount of energy transmitted by surface unit) is inversely proportional to the scan speed [51]. Thus, a lower scan speed is associated with a higher energy density. This demonstrated a potential compensation of a low absorbance by a high sintering energy.

### 3.3. Characterization of the Printed SODFs:

#### 3.3.1. Differential Scanning Calorimetry

**Figure 4:**
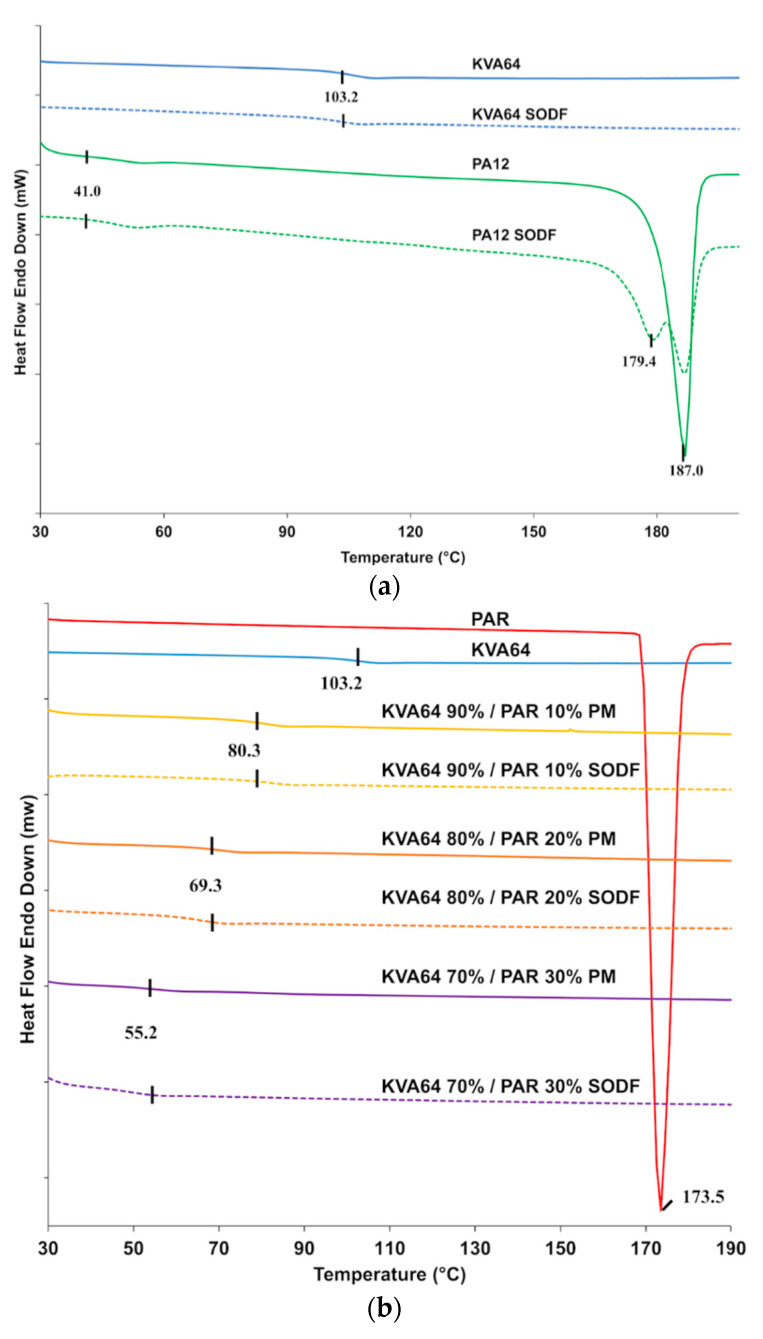
DSC curves: (**a**) KVA64 and PA12 before and after sintering (**b**) KVA64, paracetamol, physical mixtures and sintered SODFs.

DSC analysis of copovidone powder (Figure 4a) did not reveal a melting peak but only a point of inflection corresponding to the glass transition temperature (Tg = 103 °C), which is in agreement with the supplier data. These results indicate that the polymer is in an amorphous state. The curve for the KVA64 SODFs was similar indicating that sintering did not modify the plasticity of the polymer. Polyamide 12 is a semi-crystalline polymer and showed a distinctive endothermic peak characteristic of the melting temperature at 187 °C and a glass transition at 41 °C (Figure 4a). SODFs produced with PA12 showed, besides a Tg at 41 °C, two peaks around 187 °C. This is probably due the rapid cooling of the SODF’s external surface compared to the core, resulting in a heterogeneous crystallization.

From the DSC curve showed in Figure 4a, it appears that copovidone does not endure solid state transition, unlike polyamide 12, which recrystallization is mainly influenced by the post-sintering cooling rate. This may constitute an advantage for amorphous polymers like Kollidon^®^ VA64, since crystallization is an important determinant of shrinkage and dimension inaccuracy [52].

Figure 4b shows an endothermic peak for paracetamol at 173 °C, which corresponds to its melting point. However, this melting peak was not found in the different mixtures. This suggests that an amorphization of paracetamol occurs during the first DSC heating cycle conducted up to 200 °C to remove the thermal history of copovidone. During this first thermal scan, paracetamol seems to dissolve into the molten polymer explaining the absence of its characteristic melting peak in the second thermal scan. The DSC curve in the second heating cycle does not reflect the original solid state of the drug but rather its state in the solid amorphous dispersion formed *in situ*. Consequently, physical mixtures and sintered SODFs were indistinguishable from each other on DSC as both appear amorphous with a similar Tg (Figure 4b). An anterior study reported the limitations of DSC to study the solid state of drugs, which can lead to inaccurate conclusions [53]. Hence, thermal analysis should be coupled to another technique such XRPD for a proper and complete characterization of the solid state.

Figure 4b shows that glass transition temperatures are shifted to lower temperatures in both mixtures and SODFs: 80 °C, 69 °C and 55 °C, respectively for 10% PAR, 20% PAR, and 30% PAR. This evidences the plasticizing effect of paracetamol, which has already been demonstrated in HME [47].

The optimal heating temperature is highly correlated with the thermal properties of the polymer. For KVA64, it is set slightly above its glass transition temperature unlike PA12 that needs to be heated at a lower temperature than its melting point (Table 2). This printing parameter was found to be critical for the smooth running of the process as it minimizes the amount of energy required by the laser and also reduces the thermal gradient between surface temperature and sintering temperature. As previously demonstrated by Goodridge et al. [5], when bed temperature was too low edges of the sintered layers curled and were trained by the recoating blade at its passage, which prevented the binding between the superimposed sintered layers. Likewise, at high bed temperature, surrounding powder became hard and could form a “powder cake”, which also affected the rheological behavior of particles. Therefore, the temperature that guaranteed the printing of all 36 SODFs with minimum defects (no curling or powder cake) reproducibly was qualified as optimal.

**Figure 5:**
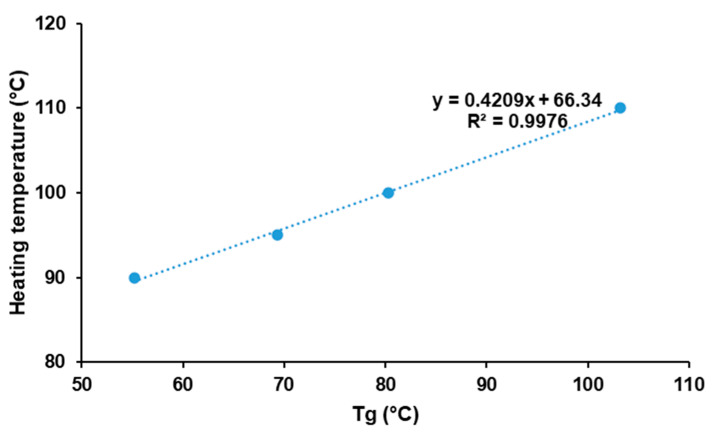
Evolution of optimal heating temperature in function of glass transition temperature.

Moreover, incorporation of paracetamol had a beneficial effect by lowering the optimal heating temperature, which is mainly attributed to the plasticizer effect of paracetamol. Figure 5 demonstrates the existence of a linear relationship between the Tg of physical mixtures and the optimal heating temperature (R^2^ = 0.9976). This suggests an interesting method to predict the optimal heating temperature for mixtures of amorphous polymers and drugs. However, those results may be specific to KVA64 and paracetamol. Hence, further studies should be conducted to assess the replicability of this method with other polymers and/or drugs.

#### 3.3.2. XRPD

**Figure 6:**
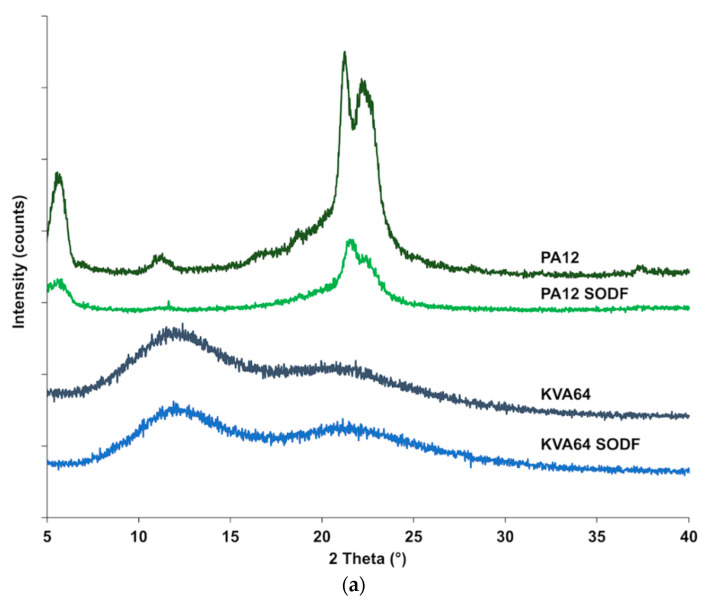

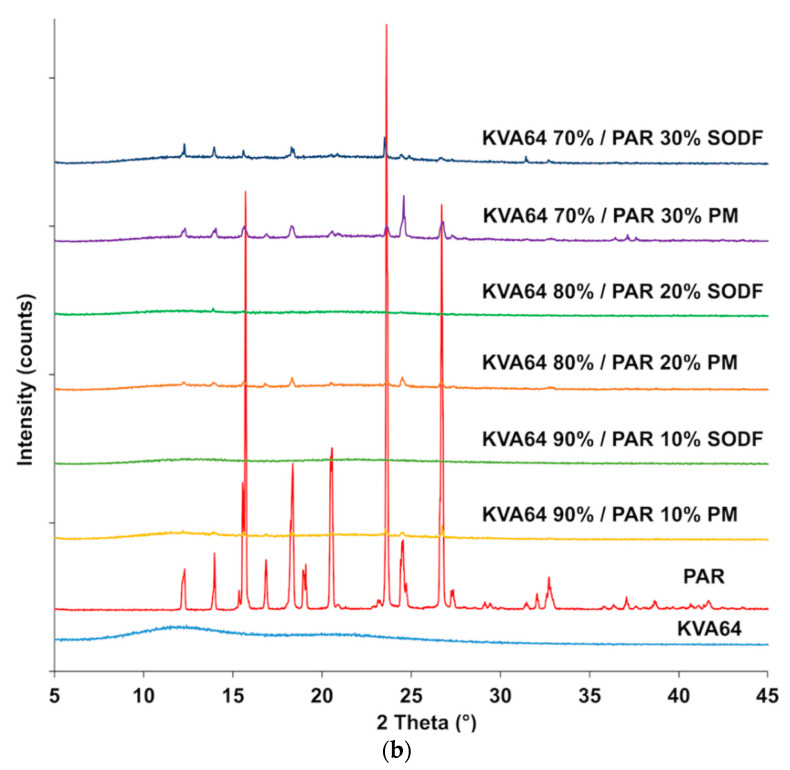
XRPD patterns: (**a**) KVA64 and PA12 before and after sintering (**b**) KVA64, paracetamol, physical mixtures and sintered solid oral dosage forms (SODFs).

X-ray powder diffraction (Figure 6a) confirmed that Kollidon^®^ VA64 is amorphous and it does not crystallize after sintering, since no crystalline peaks were distinguished after the process. The X-ray diffractogram of polyamide 12 (Figure 6a) shows characteristic diffraction peaks at 5.7°, 11.3°, 21.3°, and 22.4°, which are distinctive of a more ordered crystalline structure. Diffractogram of SODFs produced with polyamide 12 exhibited the same distinctive peaks but with a reduced intensity. This can be attributed to a decrease in crystallinity due to the sintering process that does not allow optimal crystallization upon rapid cooling. Those results corroborate the observations made by the DSC analysis.

X-ray diffractogram (Figure 6b) of the drug evidenced its distinct crystalline state, exhibiting an XRPD pattern in agreement with the standard JCPDS n° 15-3905 of paracetamol in the form-I (JCPDS: Joint Committee on Powder Diffraction Standards). Those characteristic paracetamol peaks were also present in the physical mixtures, and their intensity increased with the proportion of drug present. In the sintered SODFs (Figure 6b), the crystalline peaks are reduced or even disappeared at low drug loadings (10% and 20%), indicating the amorphization of the paracetamol when it is dissolved into the molten polymer. This confirms the ability of SLS to produce solid amorphous dispersions [7,17,18]. However, the XRPD analysis of the formulation 70% KVA64/30% PAR shows that only partial amorphization occurred and the drug remained essentially crystalline, which is in agreement with previous findings that suggests that high drug loadings hinder amorphization in SLS [10].

#### 3.3.3. Properties of the Printed SODFs

**Figure 7:**
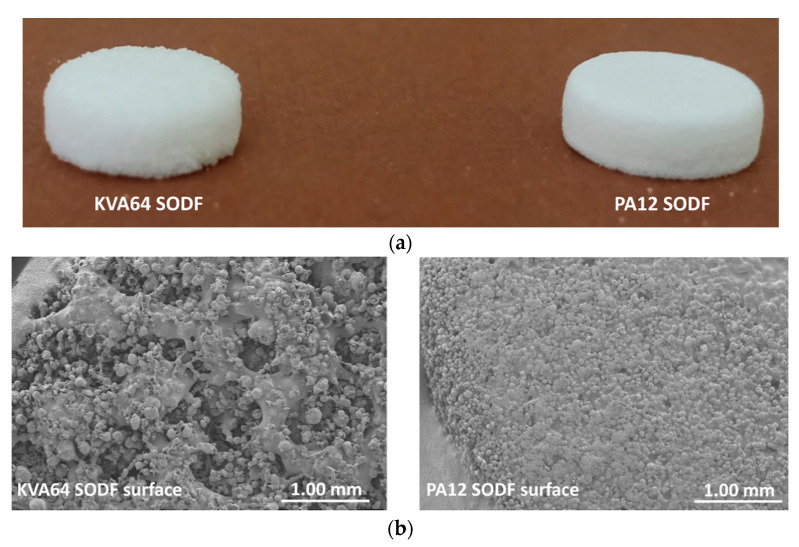
General aspect of KVA64 and PA12 SODFs printed at optimal parameters: (**a**) Images of the SODFs, (**b**) SEM images of the SODF surfaces (Magnification ×30).

In general, SODFs produced with copovidone (KVA64 and other printable mixtures) were visually assessed of inferior quality than those printed with PA12 (Figure 7a). The rough surfaces and the less accurate shape of copovidone’s SODFs were mainly due to the presence of larger proportion of coarse particles and especially when paracetamol was added (Supplementary data: Figure S1). SEM images of the SODF surfaces (Figure 7b) showed the presence of non-sintered particles around both SODFs, which explains the powdery aspect of the SODFs. Besides, they confirmed the macroscopic observations demonstrating that the PA12 SODFs were more regular on their surface than the KVA64 SODFs. These visual defects may weaken treatment compliance, but the powdery aspect of the sintered SODFs reminds more of the SODFs produced by more classical manufacturing processes such direct compression, comparing SODFs obtained by FDM [19].

**Figure 8:**
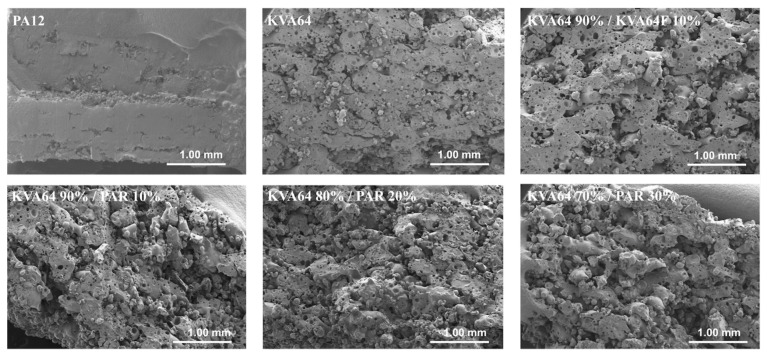
SEM images of the SODFs vertical sections (Magnification ×30).

The observation of the vertical sections of printed SODFs on SEM (Figure 8) permitted to appreciate their pore structure. KVA64 SODFs exposed a higher porosity compared to PA12 SODFs, which appeared much denser. Furthermore, SODFs printed with the mixture KVA64 90%/KVA64F 10% presented an increased porosity compared to the KVA64 SODFs (Figure 8). The same observations can be made for SODFs printed with the mixtures of KVA64 and PAR. This could be explained by the reduction of compactness after the addition of fine particles of copovidone or paracetamol powder as the decrease in the bulk density values demonstrated (Table 4). Low compactness of powder is associated with a decrease in thermal absorptivity, which results in a poor densification of the printed part and an increase of porosity [26]. Moreover, for the case of paracetamol the reduced density can be correlated with its non-absorbance at 10.6 µm. Even though Fina et al. [7] reported that high loadings of paracetamol are associated with less porosity, the use of a different polymer, a different laser beam, and other printing parameters make this study not comparable to the work here presented. Since the particles of paracetamol do not absorb at the laser’s wavelength, they are not melted by thermal absorptivity but rather by thermal conductivity, which is also known to be affected by poor packing behavior [54]. Likewise, paracetamol, which has a higher melting point (173 °C), does not dissolve completely into the rubbery polymer and part of the drug remains unmodified, especially when it is highly loaded as the partial amorphization observed on XRPD proves it (Figure 6). Consequently, the non-melted particles of paracetamol space up the particles of copovidone and hinder the formation of a continuous melting pool, which increases the porosity. Nonetheless, SEM images (Figure 8) did not allow to distinguish a difference in porosity between the different mixtures of KVA64 and PAR, which could be associated to their similar compactness (BD = 0.34 g/cm^3^).

Concerning SODF’s size, SODFs printed with KVA64 and its mixtures exceeded the input dimensions by 20–30% (Table 5). At the opposite, SODFs printed with PA12 were matching more the established CAD’s design. This could be explained by the presence of coarse particles that exceed the layer thickness input on the G-code, resulting in an overall height superior to the designed value. A previous study [55] established that a D90 (diameter where 90% of the distribution has a smaller particle size) much smaller than the layer thickness is a preliminary requirement suitable for SLS, which was not satisfied in the case of KVA64 and its mixtures.

The weight of SODFs sintered with copovidone varied from 183 to 200 mg (Table 6). We can notice that the introduction of paracetamol in the formulations decreases the SODF weight, which can be correlated with the lower compactness of the mixtures compared to pure KVA64.

Regarding SODF’s hardness (Table 6), PA12 SODFs were ductile and did not break when submitted to an increasing horizontal force but only deformed. As their hardness often exceeded the machine superior limits. Oppositely, KVA64 SODFs were brittle and exhibited a mean hardness of 89.3 N. Here, the solid state of the polymer is determinant with objects printed with semi-crystalline polymers such as polyamide 12 exhibiting better mechanical properties [56]. Moreover, SODFs printed with 90% KVA64/10% KVA64F presented similar hardness than KVA64 SODFs (Table 6), whereas the hardness of SODFs containing paracetamol was considerably reduced (almost 50%). This could be due to a poor interparticular cohesion, which resulted from an increased porosity as explained above.

All SODFs disintegrated in less than 15 min (Table 6) which is in agreement with the recommended disintegration time for uncoated tablets by the European Pharmacopeia [57]. Disintegration times were lower when paracetamol was included, and disintegration accelerated with the drug loading. During the disintegration assay, SODFs containing copovidone gelled and eroded in block until complete dissolution. In SODFs printed with both KVA64 and PAR, the drug that was not dispersed into the molten polymer dissolved in the water during the disintegration assay and created channels disaggregating the SODF into many fragments which gelled and eroded individually. The decrease in disintegration time could also be correlated with the increased porosity of SODFs and low compactness of the powders.

The possibility of drug degradation due to the considerably high energy of the CO_2_ laser was a major concern for this study. UHPLC analysis of SODFs printed with KVA64 and PAR at different loadings, revealed only one peak corresponding to the paracetamol at a retention time of 1.26 min. Drug content was also evaluated, and the results (Table 6) were in agreement with the theoretical percentages of paracetamol (10%, 20% and 30%). This proves that the CO_2_ laser did not denature paracetamol at the applied printing parameters (scan speed set at 25,000 pps and laser power set at 25%). More drastic printing conditions associated with a higher sintering energy could, however, degrade the drug. Hence, printing parameters should be optimized to ensure sintering while preserving the integrity of the drug. Moreover, SEC-MALS analysis revealed no difference in average molecular weight between the copovidone present in the native powder and the polymer contained in the sintered KVA64 placebo SODF (data not shown). This demonstrates that the polymer was not degraded by the sintering process, making it a safe and suitable polymeric carrier for pharmaceutical applications intended by CO_2_ laser sintering.

**Figure 9:**
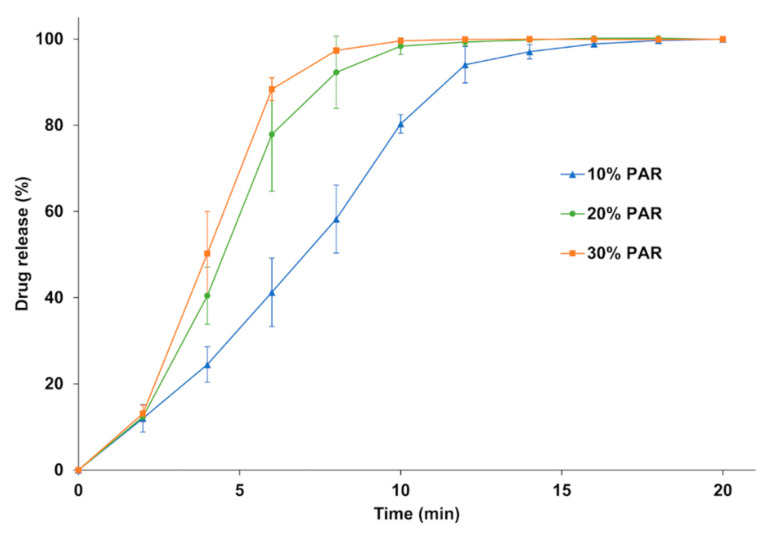
Dissolution profile of the SODFs sintered with KVA64 & PAR at different loadings.

Dissolution tests (Figure 9) were carried out to evaluate the dissolution rate of the SODFs printed with KVA64/PAR depending on the drug loading. For all three formulations, 85% of drug release was achieved within 15 min which make these SODFs suitable for immediate release according to the guidelines of the European Medicines Agency [58]. The dissolution rate increased with drug loading and evolved in the sense of disintegration time. Complete dissolution was achieved at 12, 14 and 18 min respectively for 30%, 20%, and 10% of paracetamol. The drug release rate from amorphous solid dispersions prepared with copovidone was previously demonstrated to be controlled solely by the polymer erosion mechanism [59]. Hence, by augmenting drug loading, the disintegration rate increases, exposing a larger surface area to the dissolution medium, which increases the erosion rate and accelerates drug release. Also, by reducing the proportion of KVA64, viscosity of the medium decreases which may accelerate erosion and drug release [6,60,61].

## 4. Conclusions

In this study, the production of solid oral dosage forms with copovidone and paracetamol by SLS using a CO_2_ laser was demonstrated for the first time. The ability of KVA64 to absorb at the laser’s wavelength (10.6 µm) make it suitable for SLS, and the addition of an absorbance enhancer was not necessary. Furthermore, UHPLC analysis confirmed that no drug degradation occurred during sintering despite the relatively high power of the laser. This opens a new area of research in the use of this type of printer for the preparation of SODFs. However, more thermosensitive drugs could be affected by the CO_2_ laser and their degradation should be evaluated in further studies.

Flowability was found to be critical for the process and was mainly dependent on the morphology and granulometry of the particles. Hence, in the preparation of formulations for SLS, not only the grade of polymer (KVA64) have to be chosen correctly but also the grade of the API (PAR). Mixtures of KVA64 and PAR presented lower compactness compared to the reference material (PA12), which resulted in mediocre mechanical properties. However, if high density is usually preferable for printed parts intended for engineering, presence of porosity is more interesting for pharmaceutical applications especially for modulation of drug release. The percentage of drug was proven to have an impact on the sintering process by lowering the heating temperature of the powder due to the plasticizer effect of paracetamol. Different drug loadings also influenced the SODF properties, especially drug release.

Overall, Kollidon^®^ VA64 has potential in 3D printing techniques, and this aptitude could be considerably boosted for SLS when powder particles are matching the morphological and rheological requirements for the technology: adequate particle shape, size distribution, and most importantly, a good flowability. This confirms that critical quality attributes of raw materials need to be rethought with the advent of new pharmaceutical production processes like additive manufacturing. In order to facilitate the establishment of the SLS technology in the pharmaceutical landscape, future studies would be encouraged to explore further the material-process relationship and to optimize the feedstock’s printability with physical modifications. Nevertheless, this study suggests some predictive tools for the “sinterability” of polymeric excipients: measurement of the absorbance at the laser’s wavelength, evaluation of the compactness using bulk density and study of flowability by calculation of Hausner ratio and angle of repose. This demarche is interesting since no GMP certified SLS machine nor pharmaceutical grade feedstock are commercially available.

## Figures and Tables

**Table 1:** Composition of the different mixtures.

Mixtures	KVA64	KVA64F	PAR	PAR F
90% KVA64/10% KVA64F	90%	10%	/	/
80% KVA64/20% KVA64F	80%	20%	/	/
70% KVA64/30% KVA64F	70%	30%	/	/
90% KVA64/10% PAR	90%	/	10%	/
80% KVA64/20% PAR	80%	/	20%	/
70% KVA64/30% PAR	70%	/	30%	/
90% KVA64/10% PAR F	90%	/	/	10%
80% KVA64/20% PAR F	80%	/	/	20%
70% KVA64/30% PAR F	70%	/	/	30%

**Table 2:** Printing parameters for the different powders.

Printing Parameters	Heating Temperature (°C)	Laser Power (%)	Scan Space (pps)	Layer Thickness (mm)
PA12	165	25	45,000	0.1
KVA64	110		25,000	
90% KVA64 10% KVA64F				
90% KVA64 10% PAR	100			
80% KVA64 20% PAR	95			
70% KVA64 30% PAR	90			

**Table 3:** Mean particle size and span of the native powders and prepared mixtures.

Powder	D (4,3) (µm)	Span
KVA64	71.49 ± 0.96	2.16 ± 0.03
KVA64F	26.00 ± 0.15	2.16 ± 0.02
PA12	63.66 ± 0.29	0.93 ± 0.04
PAR	124.66 ± 4.49	3.34 ± 0.02
PAR F	15.40 ± 0.12	2.25 ± 0.02
90% KVA64/10% KVA64F	64.20 ± 2.94	2.42 ± 0.06
80% KVA64/20% KVA64F	56.84 ± 0.76	2.63 ± 0.03
70% KVA64/30% KVA64F	51.44 ± 0.37	2.84 ± 0.02
90% KVA64/10% PAR	81.91 ± 0.28	2.32 ± 0.01
80% KVA64/20% PAR	88.87 ± 3.31	2.59 ± 0.05
70% KVA64/30% PAR	92.46 ± 1.89	2.84 ± 0.04
90% KVA64/10% PAR F	68.28 ± 1.65	2.28 ± 0.02
80% KVA64/20% PAR F	63.20 ± 2.26	2.54 ± 0.06
70% KVA64/30% PAR F	58.38 ± 0.70	2.77 ± 0.05

D (4,3): mean particle size.

**Table 4:** Bulk density, tapped density, Hausner ratio, angle of repose, and flow property of the native powders and prepared mixtures.

Powder	BD (g/cm^3^)	TD (g/cm^3^)	HR	AOR (°)	Flow Property *
KVA64	0.38 ± 0.00	0.48 ± 0.01	1.25 ± 0.02	37.73 ± 0.93	Fair
KVA64F	0.12 ± 0.00	0.19 ± 0.01	1.57 ± 0.10	/	Very poor
PA12	0.48 ± 0.01	0.58 ± 0.01	1.19 ± 0.04	34.00 ± 0.17	Good
90% KVA64/10% KVA64F	0.35 ± 0.01	0.43 ± 0.00	1.24 ± 0.02	39.17 ± 1.62	Fair
80% KVA64/20% KVA64F	0.30 ± 0.01	0.40 ± 0.00	1.33 ± 0.05	/	Passable
70% KVA64/30% KVA64F	0.26 ± 0.01	0.35 ± 0.01	1.36 ± 0.02	/	Poor
90% KVA64/10% PAR	0.34 ± 0.00	0.49 ± 0.01	1.43 ± 0.03	36.84 ± 0.06	Fair
80% KVA64/20% PAR	0.34 ± 0.00	0.51 ± 0.01	1.50 ± 0.03	39.67 ± 0.55	Fair
70% KVA64/30% PAR	0.34 ± 0.00	0.52 ± 0.01	1.53 ± 0.03	40.67 ± 0.97	Fair
90% KVA64/10% PAR F	0.31 ± 0.01	0.48 ± 0.00	1.56 ± 0.04	/	Very poor
80% KVA64/20% PAR F	0.28 ± 0.00	0.49 ± 0.01	1.79 ± 0.04	/	Very, very poor
70% KVA64/30% PAR F	0.26 ± 0.00	0.49 ± 0.02	1.85 ± 0.06	/	Very, very poor

BD: bulk density, TD: tapped density, HR: Hausner ratio, AOR: angle of repose. * Classification according to the European Pharmacopeia [31].

**Table 5:** Flat layer formation and printability of the native powders and prepared mixtures.

Powder	Flow Property	Flat Layer at the 1st Attempt	Printability
KVA64	**Fair**	YES	**YES**
KVA64F	Very poor	NO	NO
PA12	**Good**	YES	**YES**
90% KVA64/10% KVA64F	**Fair**	YES	**YES**
80% KVA64/20% KVA64F	Passable	YES	NO
70% KVA64/30% KVA64F	Poor	YES	NO
90% KVA64/10% PAR	**Fair**	YES	**YES**
80% KVA64/20% PAR	**Fair**	YES	**YES**
70% KVA64/30% PAR	**Fair**	YES	**YES**
90% KVA64/10% PAR F	Very poor	NO	NO
80% KVA64/20% PAR F	Very, very poor	NO	NO
70% KVA64/30% PAR F	Very, very poor	NO	NO

**Table 6:** Dimensions, weight, hardness, disintegration time and drug content of printed SODFs.

SODF	PA12	KVA64	90% KVA64 10% KVA64F	90% KVA64 10% PAR	80% KVA64 20% PAR	70% KVA64 30% PAR
T (mm)	3.20 ± 0.02	4.15 ± 0.09	4.08 ± 0.20	3.99 ± 0.14	3.72 ± 0.06	3.76 ± 0.09
D (mm)	10.00 ± 0.12	10.51 ± 0.20	10.44 ± 0.08	10.60 ± 0.11	10.52 ± 0.13	10.50 ± 0.12
W (mg)	196.00 ± 6.33	200.80 ± 4.66	192.90 ± 9.05	188.40 ± 2.63	183.80 ± 3.65	184.80 ± 1.99
H (N)	>500	89.32 ± 12.94	85.57 ± 9.48	55.59 ± 4.28	47.93 ± 7.19	47.18 ± 4.36
DT (s)	/	328.0 ± 67.0	262.0 ± 39.0	223.0 ± 8.1	134.0 ± 6.6	119.0 ± 5.0
DC (%)	/	/	/	10.03 ± 0.82	20.18 ± 0.75	29.81 ± 0.49

T: thickness–D: diameter–W: weight—H: hardness–DT: disintegration time–DC: drug content.

## Data Availability

Data is contained within the article or supplementary material.

## Supplementary Materials

The following are available online at https://www.mdpi.com/1999-4923/13/2/160/s1, Figure S1: Particle size distribution of powders: (a) native powders, (b) mixtures of KVA64 and KVA64F, (c) mixtures of KVA64 and PAR, (d) mixtures of KVA64 and PAR F. Figure S2: FTIR spectra of mixtures KVA64/PAR.
