# Supplementary material for: Selective Laser Sintering of Solid Oral Dosage Forms with Copovidone and Paracetamol Using a CO2 Laser
Source: Pharmaceutics. 2021 Jan 26;13(2):160. doi: 10.3390/pharmaceutics13020160 (PMC7911894; doi:10.3390/pharmaceutics13020160)
Supplement: Supplementary file 1 [file pharmaceutics-13-00160-s001.pdf]

pharmaceutics

MDPI

Article

Supplementary Materials: Selective Laser Sintering of Solid Oral Dosage Forms with Copovidone and Paracetamol Using a CO_2 Laser

Yanis A. Gueche, Noelia M. Sanchez-Ballester, Bernard Bataille, Adrien Aubert, Laurent Leclercq, Jean-Christophe Rossi and Ian Soulairol

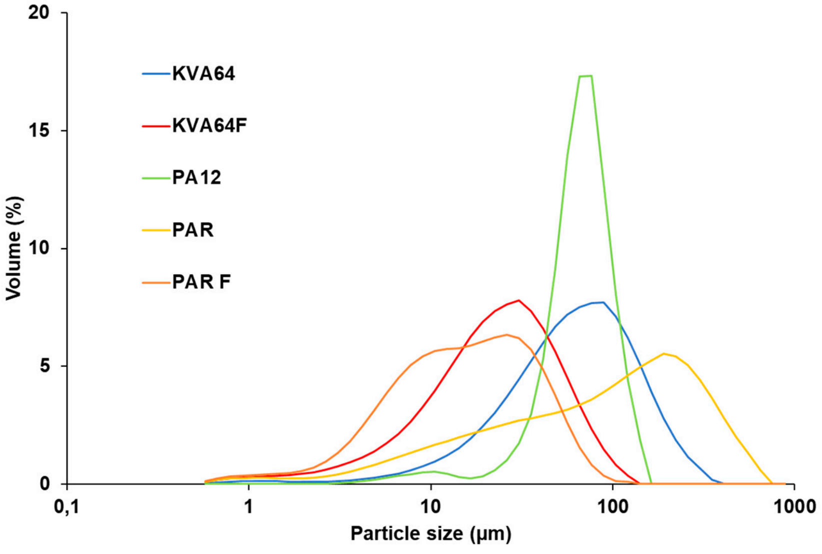

(a)

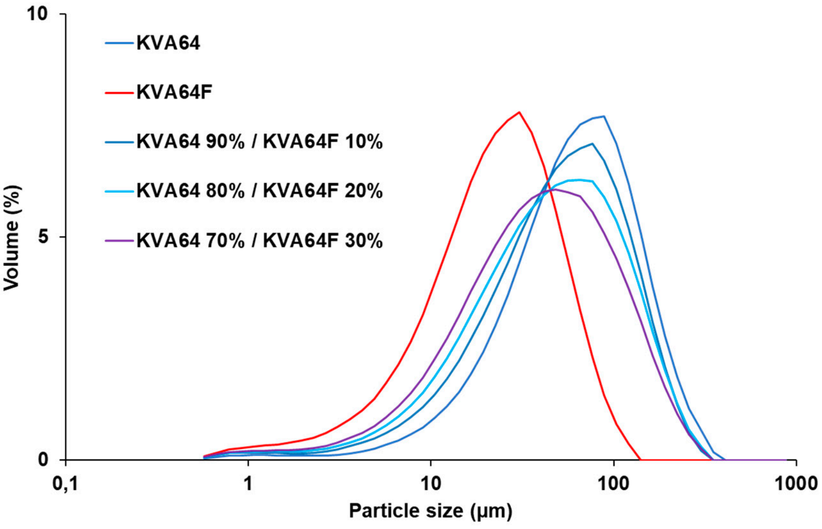

(b)

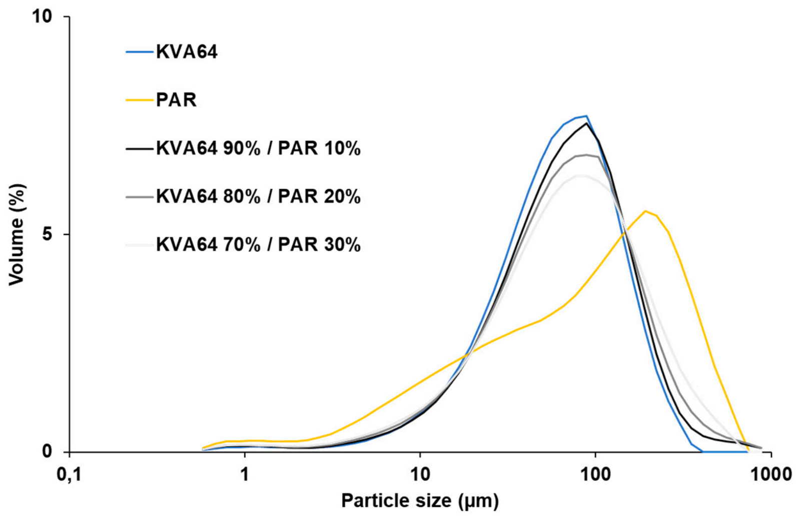

(c)

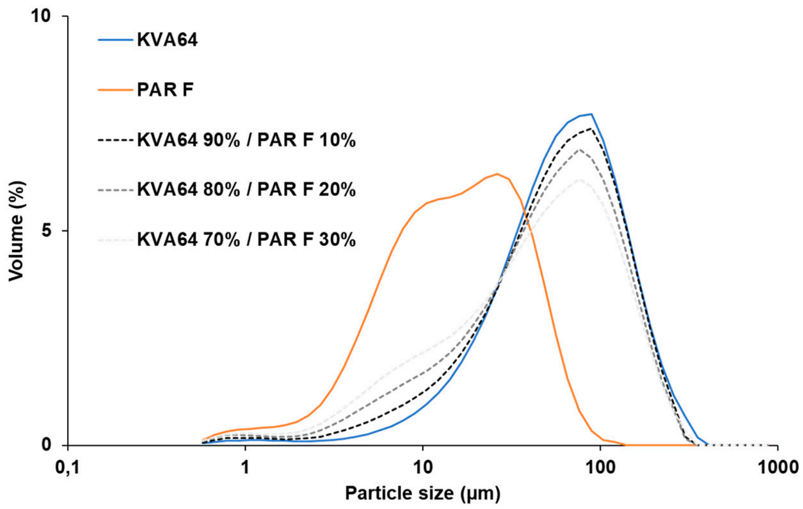

(d)

Figure S1. Particle size distribution of powders: (a) native powders, (b) mixtures of KVA64 and KVA64F, (c) mixtures of KVA64 and PAR, (d) mixtures of KVA64 and PAR F.

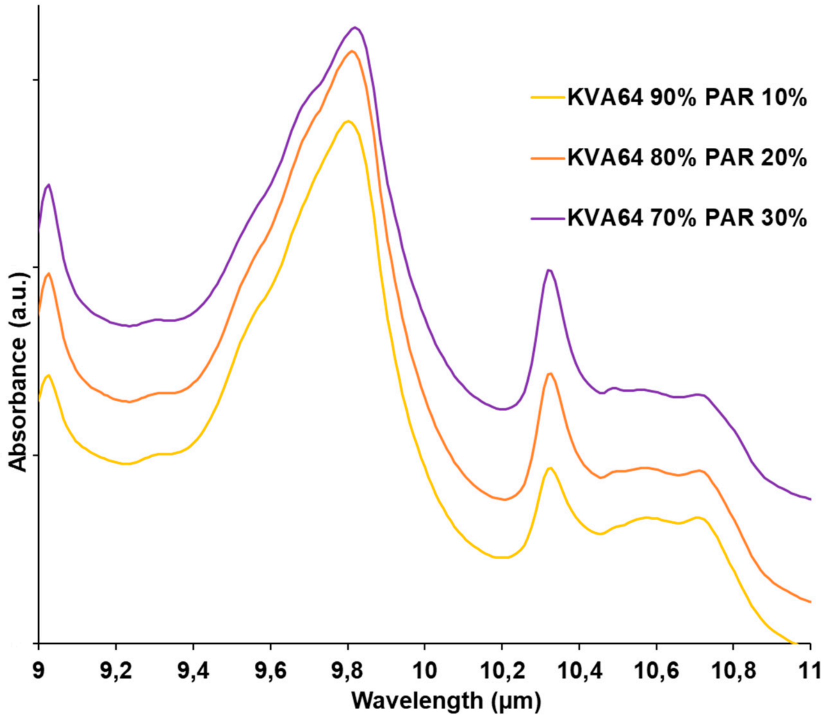

Figure S2. FTIR spectra of mixtures KVA64 / PAR.
